# Co-encapsulation of curcumin and quercetin with zein/HP-β-CD conjugates to enhance environmental resistance and antioxidant activity

**DOI:** 10.1038/s41538-023-00186-2

**Published:** 2023-06-14

**Authors:** Chao Qiu, Zhiheng Zhang, Xiaojing Li, Shangyuan Sang, David Julian McClements, Long Chen, Jie Long, Aiquan Jiao, Xueming Xu, Zhengyu Jin

**Affiliations:** 1 State Key Laboratory of Food Science and Resources, School of Food Science and Technology, International Joint Laboratory on Food Safety, Collaborative Innovation Center of Food Safety And Quality Control in Jiangsu Province, Jiangnan University, Wuxi, Jiangsu 214122 China; 2 College of Light Industry and Food Engineering, Nanjing Forestry University, Nanjing, Jiangsu 210037 China; 3 Zhejiang-Malaysia Joint Research Laboratory for Agricultural Product Processing and Nutrition, Key Laboratory of Animal Protein Food Deep Processing Technology of Zhejiang Province, College of Food and Pharmaceutical Sciences, Ningbo University, Ningbo, 315832 China; 4 Department of Food Science, University of Massachusetts, Amherst MA, 01060 USA

**Keywords:** Nanoparticles, Drug delivery

## Abstract

In this study, composite nanoparticles consisting of zein and hydroxypropyl beta-cyclodextrin were prepared using a combined antisolvent co-precipitation/electrostatic interaction method. The effects of calcium ion concentration on the stability of the composite nanoparticles containing both curcumin and quercetin were investigated. Moreover, the stability and bioactivity of the quercetin and curcumin were characterized before and after encapsulation. Fluorescence spectroscopy, Fourier Transform infrared spectroscopy, and X-ray diffraction analyses indicated that electrostatic interactions, hydrogen bonding, and hydrophobic interactions were the main driving forces for the formation of the composite nanoparticles. The addition of calcium ions promoted crosslinking of the proteins and affected the stability of the protein–cyclodextrin composite particles through electrostatic screening and binding effects. The addition of calcium ions to the composite particles improved the encapsulation efficiency, antioxidant activity, and stability of the curcumin and quercetin. However, there was an optimum calcium ion concentration (2.0 mM) that provided the best encapsulation and protective effects on the nutraceuticals. The calcium crosslinked composite particles were shown to maintain good stability under different pH and simulated gastrointestinal digestion conditions. These results suggest that zein–cyclodextrin composite nanoparticles may be useful plant-based colloidal delivery systems for hydrophobic bio-active agents.

## Introduction

Plant-based polyphenolic compounds have attracted widespread interest as bio-active ingredients in foods because of their diverse range of potentially beneficial physiological functions, such as antioxidant, antimicrobial, anti-inflammatory, anti-glycemic, anti-obesity, anti-atherosclerosis, and anticancer activities^1^. However, many polyphenolic compounds, such as curcumin (Cur) and quercetin (Que), are chemically labile hydrophobic substances that are susceptible to chemical degradation and have low water solubility, which reduces their stability in foods and leads to a low bioavailability after ingestion^2,3^. In addition, lipophilic polyphenols are easy to interact with digestive enzymes during gastrointestinal digestion, thus affecting the absorption efficiency of polyphenol compounds^4,5^. This greatly limits their practical application as nutraceuticals in plant-based foods and beverages. An effective strategy to overcome these issues is to encapsulate the polyphenols in edible nanocarriers^6^. These nanoparticles can be created from food-grade ingredients, such as proteins, polysaccharides, phospholipids, and lipids. Biopolymer-based nanocarriers have gained strong interest because their compositions and structures can easily be manipulated to create delivery systems with different functional attributes^7,8^.

Zein is a natural macromolecule derived primarily from starch and ethanol production byproducts. It is low-cost, environmentally friendly, and biocompatible, and has been approved for use by the US Drug Administration^9^. Zein has a unique amino acid composition (more than half of the hydrophobic amino acids) so that it can only be dissolved in a certain concentration of ethanol aqueous solution (55–90%), but not in water or absolute ethanol^10^. This also allows zein to encapsulate the above-mentioned hydrophobic bio-active compounds by a simple antisolvent precipitation method. Nevertheless, the utilization of zein nanoparticles as colloidal delivery systems still has some drawbacks. The nanoparticles tend to aggregate when exposed to certain ionic strength, pH, and temperature conditions^11^. This is mainly because of the strong hydrophobic attraction between them. As a result, it is important to ensure there are strong repulsive interactions, such as steric or electrostatic repulsion, between the zein nanoparticles to counteract the attractive hydrophobic interactions. Several studies have reported that polysaccharide coatings can be used to reduce the aggregation of zein nanoparticles, such as pectin^12^, soybean polysaccharide^13^, carrageenan^14^, fucoidan^15^, chitosan^16^, and gum arabic^17^. These polysaccharides adsorb to the surfaces of the zein nanoparticles and form a thick, charged interfacial layer that generates strong steric and electrostatic repulsion. Even so, many kinds of polysaccharide-coated zein nanoparticles have still been reported to be susceptible to aggregation when environmental conditions are changed^18^.

Calcium ions could be used to regulate the properties of complex colloidal particles through electrostatic screening or linking effects^19^. In the case of proteins, cationic calcium ions can bind to anionic groups on the polypeptide chains, thereby altering the overall electrical characteristics. Moreover, in certain concentration ranges, cationic calcium ions can act as salt bridges between anionic groups on different molecules. In biology, the presence of calcium ions is known to alter the structure of some proteins and affect their biological properties^20^. In the food industry, most of the previous research on the effects of calcium ions on the formation and properties of protein-based nanoparticles had focused on water-soluble proteins, such as those from soybeans^21^, wheat germ^22^, and whey^23^. There have also been extensive studies on the interactions of calcium ions with polysaccharide molecules and the formation of polysaccharide-based nanoparticles, such as those consisting of pectin^24^, carrageenan^19^, and fucoidan^25^. For these reasons, we used calcium ions to cross-link zein with cyclodextrin molecules and investigated the co-encapsulation effect of zein nanoparticles on curcumin and quercetin under the dual stabilization of calcium ion cross-linking and cyclodextrin coating.

Many previous studies have focused on the effects of different types of polysaccharide molecules on the formation, stability, and properties of zein nanoparticles. Ca^2+^-induced zein/HP-β-CD composite nanoparticles were designed and prepared for the co-inclusion of hydrophobic bio-active molecules quercetin and curcumin in this study. The presence of Ca^2+^ is believed to be conducive to the improvement of the stability of composite particles, with potential effects on cell membrane channels that are conducive to the absorption of nutrient molecules, and regulate the slow-release effect of nutrient molecules. And through the Fourier transform infrared spectrum (FT-IR), fluorescence spectra (FS), X-ray diffraction (XRD), scanning electron microscope (SEM), and other means to verify the electrostatic interaction, ionic crosslinking effects, hydrogen bonding, and hydrophobic interaction exist in the formation process of composite particles, and curcumin and quercetin have success in the composite particle encapsulation. In addition, the composite system also shows good antioxidant ability and environmental stress resistance, which has a promising potential for applications in functional drinks and foods as well as biology- and medicine-related delivery systems.

## Results and discussion

### Particle size, polydispersity, and charge

**Fig. 1:**
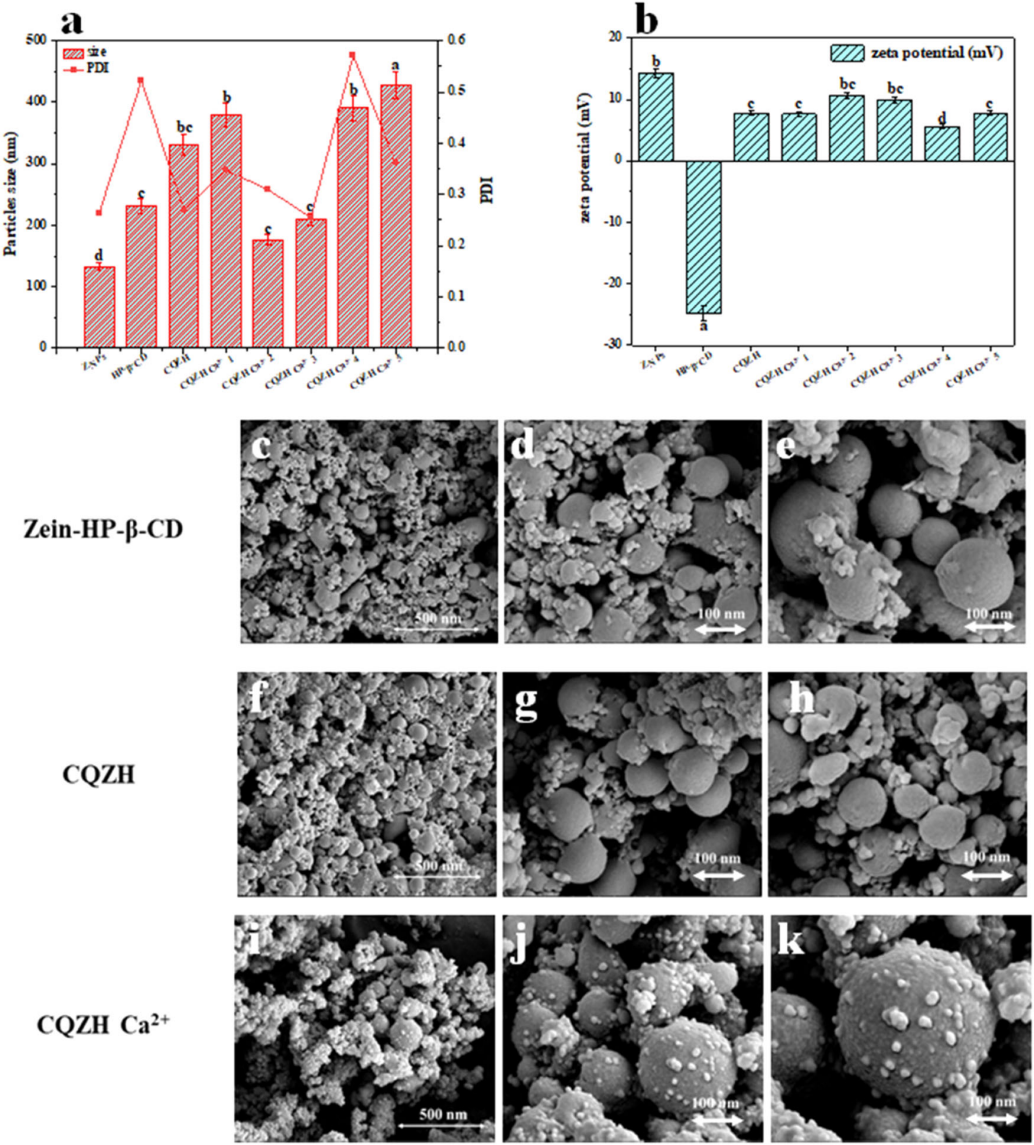
Particle size, PDI, zeta-potential, and SEM of samples. Impact of formulation on particles size (**a**), PDI (**a**), and zeta-potential (**b**) of zein, HP-β-CD, CQZH nanoparticles, and CQZH Ca^2+^ nanoparticles (1–5 mM Ca^2+^); SEM of zein-HP-β-CD (**c**: ×10 k, **d**: ×30 k, and **e**: ×50 k), CQZH (**f**: ×10 k, **g**: ×30 k, and **h**: ×50 k), and CQZH Ca^2+^ (**i**: ×10 k, **j**: ×30 k, and **k**: ×50 k). Values with different letters differ statistically (*p* < 0.05). Data were expressed as mean ± standard deviation.

The mean particle diameter, polydispersity index, and zeta potential of nanoparticles with different formulations were measured (Fig. 1). The mean particle diameter of the zein nanoparticles was about 132.8 nm, which indicates that the antisolvent precipitation method used was effective at producing small particles. The CQZH nanoparticles were larger than the pure zein ones, which may be due to the electrostatic deposition of anionic polymer molecules on the surfaces of the zein nanoparticles^26^. A similar phenomenon has been reported for the interaction of propylene glycol alginate with zein nanoparticles^27^. The presence of calcium ions clearly influences the formation and properties of the composite nanoparticles. The mean particle diameter and polydispersity index of the composite nanoparticles were relatively high at both low (1 mM) and high (4–5 mM) calcium concentrations. However, smaller, more uniform composite nanoparticles were formed at intermediate (2–3 mM) calcium concentrations. We attribute these effects to the ability of the calcium ions to promote aggregation of the nanoparticles, which results in an increase in the particle size^28^. At low calcium levels, the cationic Ca^2+^ ions may act as salt bridges that link together two or more anionic nanoparticles. At high calcium levels, the Ca^2+^ ions may reduce the magnitude of the electrostatic repulsion between the nanoparticles through electrostatic screening effects. At intermediate calcium levels, the Ca^2+^ ions bind to the anionic HP-β-CD molecules at the surfaces of the zein nanoparticles. As a result, they may promote intermolecular crosslinking of the anionic HP-β-CD molecules at the interface^20^. Presumably, the magnitude of the steric and electrostatic repulsion between the composite nanoparticles is still strong enough to inhibit extensive aggregation at intermediate calcium levels^19^.

As shown in Fig. 1b, the zeta potential value of the zein nanoparticles was +14.3 mV, and that of HP-β-CD was −24.8 mV at pH 4.0. When they were used for the co-encapsulation of curcumin and quercetin, the zeta potential value of the CQZH nanoparticles was +7.8 mV. This change indicated that the formation of the composite nanoparticles was mainly through electrostatic interactions and that the cationic zein nanoparticles dominated the overall charge^29^. When calcium ions were added, the surface potential of the composite particles became slightly more positive, which is consistent with the interaction of cationic calcium ions with anionic HP-β-CD molecules^19^.

In order to better reflect the intuitive size and morphology of nanoparticles, the SEM images of the zein-HP-β-CD, CQZH nanoparticles, and CQZH Ca^2+^ nanoparticles are shown in Fig. 1c–k. In the SEM image, the zein-HP-β-CD, CQZH, and CQZH Ca^2+^ nanoparticles appear regular and spherical, and the core-shell composite structure of zein coated by cyclodextrin can be obviously observed on the surface, and some nutrient molecules adsorbed on the surface can also be observed^19^. In addition, the size of CQZH nanoparticles and CQZH Ca^2+^ nanoparticles range from 100 to 200 nm, which is consistent with the results obtained in DLS. These results indicate that a core-shell composite nanoparticle based on zein has been successfully prepared and can be used for the encapsulation, protection, and delivery of nutrient molecules such as curcumin and quercetin.

### Encapsulation properties

**Fig. 2:**
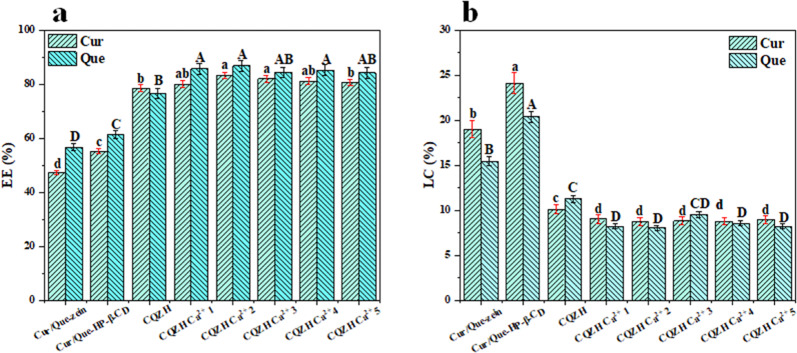
EE and LC of samples. Effect of Ca^2+^ concentration on EE (**a**) and LC (**b**) of Cur and Que in composite nano-systems. Values with different letters are statistically different (*p* < 0.05). Data were expressed as mean ± standard deviation.

The encapsulation efficiency and loading capacity of bio-active agents are important indicators of the potential efficacy of nano-delivery systems. As shown in Fig. 2, the encapsulation efficiencies of curcumin and quercetin were 47.3% and 56.7% in the zein nanoparticles, and 55.2% and 61.3% in the HP-β-CD nanoparticles, respectively. In CQZH composite nanoparticles, the encapsulation rates of curcumin and quercetin reached 78.57% and 76.55%, respectively. These results show that the encapsulation efficiencies of both nutraceuticals were significantly better in the composite nanoparticles. This effect may be due to stronger non-covalent interactions between the nanoparticles and the nutraceuticals in the composite systems and/or due to the bigger volume available to incorporate the nutraceuticals in the presence of a cyclodextrin shell^19^. The addition of calcium ions further improved the encapsulation efficiency of both nutraceuticals in the composite nanoparticles. This indicates that the added calcium ions can combine with hydroxypropyl beta-cyclodextrin and/or zein, thus changing their structures and/or interactions in a manner that increases the number of bio-active molecules that can be incorporated into the nanoparticles^30^. In contrast, the loading capacity followed the opposite trend, becoming smaller as the calcium ion concentration increased. Overall, these results suggest that a greater fraction of the nutraceuticals was incorporated into the nanoparticles in the presence of calcium, but the total quantity of nutraceuticals that could be incorporated was reduced^19^.

### FT-IR, XRD, and TG analysis

**Fig. 3:**
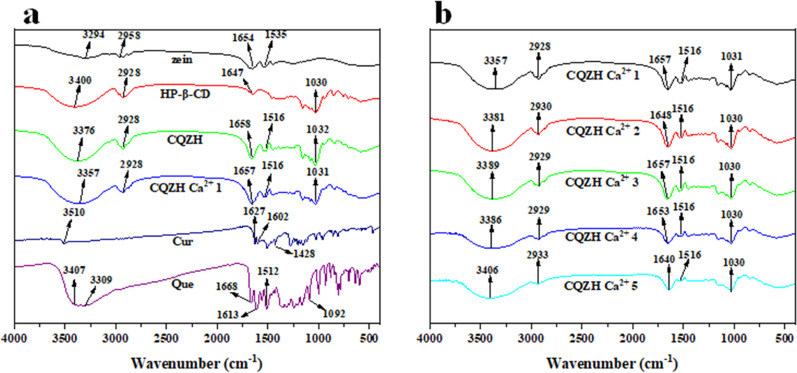
FT-IR of samples. FT-IR (**a**) of zein, HP-β-CD, zein-HP-β-CD, CQZH nanoparticles, Cur, and Que; FT-IR (**b**) of CQZH Ca^2+^ (1–5 mM Ca^2+^) nanoparticles.

The intermolecular interactions between the different constituents within the nanoparticles were investigated using FT-IR analysis (Fig. 3). For HP-β-CD, the peak at 3400 cm^−1^ is attributed to the O-H stretching vibration, and the peak at 1647 cm^−1^ is attributed to the C=O stretching vibration. For zein, the spectrum contains several peaks expected for this protein: 3294 cm^−1^ (O-H stretching vibration), 1654 cm^−1^ (amide I band), and 1535 cm^−1^ (amide II band)^19^. For the CQZH nanoparticles, the O–H stretching peaks shifted from 3294 to 3409 cm^−1^ compared to the pure zein nanoparticles, which suggested the presence of hydrogen bonding between the zein and cyclodextrin molecules. Moreover, the amide I and amide II bands shifted to 1646 and 1536 cm^−1^, respectively, indicating that electrostatic interactions also played a role in the formation of the composite nanoparticles^30^. The change in CQZH nanoparticles is essentially the same as in zein-HP-β-CD, but there are minor fluctuations. The hydrophobic interaction between quercetin, flavonoids, and zein in the composite particles is the main cause of this phenomenon^31^.

Both zein and curcumin contain a lot of hydrophobic groups, so hydrophobic interactions would also be expected to play an important role in the formation of the nanoparticles. The peaks of pure curcumin observed at 3510 cm^−1^, and pure quercetin at 3407 cm^−1^ disappeared in the CQZH composite nanoparticles, suggesting that the nutraceuticals were incorporated into the nanoparticles in an amorphous for^31^. With the addition of calcium ions, the hydroxyl vibrational peak shifted from 3376 to 3357 cm^−1^, indicating that low concentrations of calcium ions affect the hydrogen bond between zein and hydroxypropyl beta-cyclodextrin. This change became more pronounced with increasing calcium concentration. In addition, the hydrophobic interaction between the protein and the hydroxypropyl beta-cyclodextrin was seen to be further altered at increasing calcium levels^19^ (Fig. 3b).

**Fig. 4:**
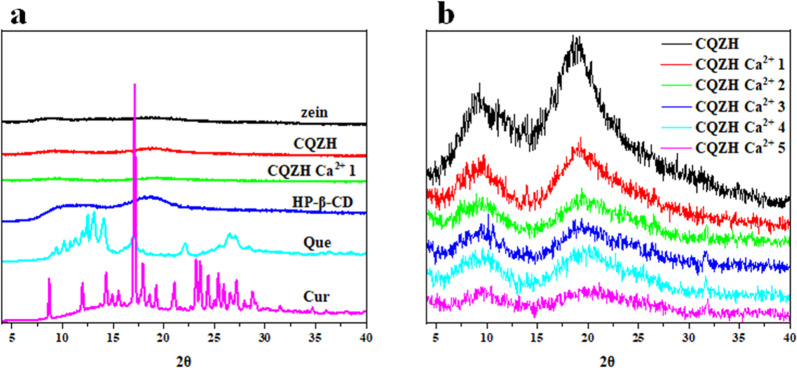
XRD of samples. XRD (**a**) of zein, HP-β-CD, zein-HP-β-CD, CQZH nanoparticles, Cur, and Que; XRD (**b**) of CQZH Ca^2+^ (1–5 mM Ca^2+^) nanoparticles.

Information about the physical state of the nanoparticles was obtained using X-ray diffraction analysis of the samples (Fig. 4). The XRD patterns of the pure curcumin and quercetin powders contained multiple sharp peaks, which indicated that they were in a crystalline state^19^. The HP-β-CD powder exhibited two broad diffraction peaks at 11° and 18.2°, while the zein powder exhibited two broad diffraction peaks at 8.1° and 18.8°, which suggested that they were both in an amorphous form^19^. The XRD patterns of all composite nanoparticles were very similar and consisted of broad peaks consistent with an amorphous form. The peaks of the composite nanoparticles were slightly shifted to 9.3° and 18.1° compared to the pure zein. In zein-HP-β-CD composite particles, the wide diffraction peaks of HP-β-CD and zein disappear, indicating that zein and HP-β-CD formed complex structures that covered and/or destroyed their own crystalline structures and finally formed amorphous composite particles^19^. Moreover, the intensity of the diffraction peaks for the composite particles gradually decreased with increasing calcium concentration. These results suggest that there were some changes in the structures of the composite nanoparticles induced by the presence of the calcium ions. No sharp diffraction peaks were observed in any of the nutraceutical-loaded composite nanoparticles, suggesting that they were present in an amorphous form^20^. These results are similar to those observed in the FT-IR atlas, and they verify the successful encapsulation of the nutrient molecules in the composite particles.

**Fig. 5:**
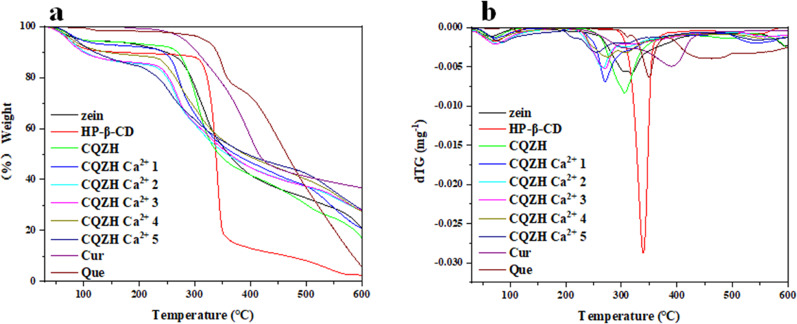
TG and dTG curves of samples. TG (**a**) and dTG (**b**) curves of zein, HP-β-CD, zein-HP-β-CD, CQZH nanoparticles, Cur, Que, and CQZH Ca^2+^ (1–5 mM Ca^2+^) nanoparticles.

In general, the overall mass versus temperature profiles of all the samples measured by thermogravimetric analysis showed similar patterns, with a slight decrease from 30 and 250 °C followed by a more substantial decrease at higher temperatures (Fig. 5). The slight mass loss observed at lower temperatures was mainly attributed to the evaporation of water, while the larger mass loss at higher temperatures was mainly attributed to thermal degradation of curcumin, quercetin, zein, and/or cyclodextrin. The maximum thermal degradation temperature of the composite nanoparticles was lower than that of zein and HP-β-CD nanoparticles, indicating that complexation improved the thermal stability of the system^30^. The maximum thermal degradation temperature of QCZH was close to that of zein-HP-β-CD, but when the temperature was increased to 250 °C, the mass loss of QCZH was significantly lower than that of zein-HP-β-CD, mainly because the core-shell nanostructure containing nutrient molecules had a tighter binding effect, which was consistent with the results reported in the study of Lai et al.^32^.

### Fluorescence, ultraviolet, and circular dichroism spectroscopy

Supplementary Fig. 1a–c shows the fluorescence intensity spectra of zein for the different nanoparticle formulations. The maximum fluorescence intensity of the zein-HP-β-CD nanoparticles was greater than that of the zein ones (Supplementary Fig. 1a), which can be attributed to an interaction between the HP-β-CD and zein molecules. Previous studies suggest that cyclodextrins can promote conformational changes in zein that expose hydrophobic amino acid residues to the surrounding water, thereby altering the fluorescence emission spectra^33^. The addition of quercetin and curcumin to the zein nanoparticles caused fluorescence quenching of the protein, which is consistent with previous studies^19,30^. At concentrations of Ca^2+^ less than 2.0 mM, the fluorescence intensity of the composite nanoparticles increased slightly, indicating that Ca^2+^ promoted some conformational changes in the zein. Presumably, the calcium ions caused a conformational change in the protein that increased the number of non-polar groups exposed to the surrounding water. When the Ca^2+^ concentration was increased to 2.0 mM, significant fluorescence quenching was observed. The light scattering results discussed earlier showed that the particle size increased significantly at higher Ca^2+^ concentrations. Nanoparticle aggregation may have led to fluorescence quenching by reducing the number of non-polar groups exposed to water^30^. Similar changes were observed in the synchronous fluorescence spectra of the tryptophan and tyrosine residues (Supplementary Fig. 1b, c).

The dependence of the UV absorption intensity of zein on nanoparticle composition is shown in Supplementary Fig. 1d. Like other proteins, zein has a weak absorption peak around 280 nm, which is related to the fact that it contains amino acids with phenolic groups (like tryptophan and tyrosine) that can absorb ultraviolet light in this range. After curcumin and quercetin were introduced, the intensity of the absorption peak shifted to around 370 nm, which can be attributed to the fact that these nutraceuticals also contain functional groups that can absorb UV–visible radiation. When the zein nanoparticles were combined with the HP-β-CD, there was only a slight change in the absorption spectra. The absorption peak at 370 nm blue-shifted to around 360 nm after calcium ions were added, indicating that they may have altered the electrostatic interactions between the zein and HP-β-CD molecules^34^. For instance, the cationic calcium ions may have competed with the cationic groups on the zein molecules for the anionic groups on the HP-β-CD molecules.

The impact of nanoparticle composition on the secondary structure of the zein was analyzed using circular dichroism in the wavelength range from 260 to 190 nm (Supplementary Fig. 2). The secondary structure of the zein was determined by analysis of the CD spectra using computer software. The α-helix content of the nutraceutical-loaded zein nanoparticles (Cur-Que-zein) was slightly higher (13.7%) than that of the pure zein nanoparticles (11.7%), indicating that incorporation of the polyphenols only caused small changes in the secondary structure of the protein^20^. In the presence of HP-β-CD, the α-helix content of the nutraceutical-loaded nanoparticles decreased to 11.1%, showing that there was also only a small change in the secondary structure after cyclodextrin was added^35^. The α-helix content decreased slightly as the calcium ion concentration was increased from 1 to 4 mM, but then increased slightly when the calcium ion concentration was further increased to 5 mM. These results suggest that the protein structure was somewhat affected by calcium ion concentration, which may have been because the binding of the multivalent cations alters the electrostatic interactions in the system^27^.

### Antioxidant activity

**Fig. 6:**
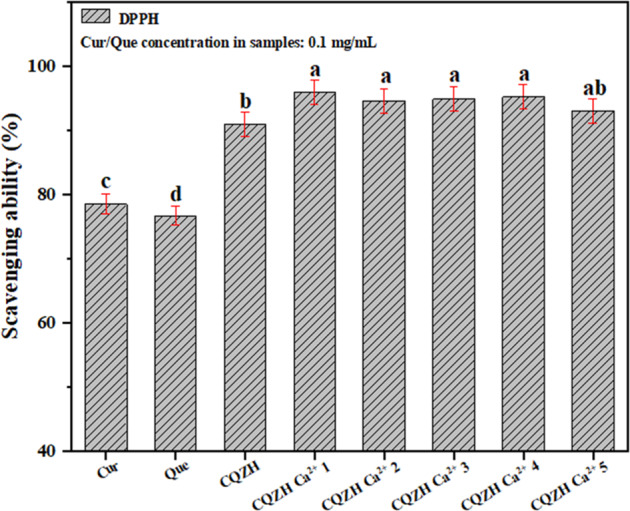
The DPPH radical scavenging rate of quercetin before and after encapsulation of different samples. Values with different letters are statistically different (*p* < 0.05). Data were expressed as mean ± standard deviation.

The antioxidant activity of polyphenol-loaded nanoparticles with different formulations was determined (Fig. 6). The free radical scavenging activity of curcumin and quercetin alone were 78.5% and 76.7%, respectively. In contrast, the free radical scavenging activity of the combined polyphenols in the composite nanoparticles was significantly enhanced (91.0%), indicating that there was a synergistic effect between the encapsulated quercetin and curcumin. The composite nanoparticles would have enhanced the dispersibility of the hydrophobic curcumin and quercetin molecules in the aqueous solutions, thereby allowing them to interact more easily with the free radicals generated in the surrounding water phase^19,31^. The presence of the calcium ions in the composite nanoparticles further enhanced the free radical scavenging ability of the polyphenols (93.1–96.0%). This is due to the fact that calcium ions can improve the encapsulation efficiency of polyphenols by improving the stability of composite particles, further enhancing the dispersibility of polyphenols in water, and increasing the contact area with free radicals^31^. These results suggest that incorporating calcium into the composite nanoparticles may be an effective means of increasing their biological activity.

### Stability of composite nanoparticles

**Fig. 7:**
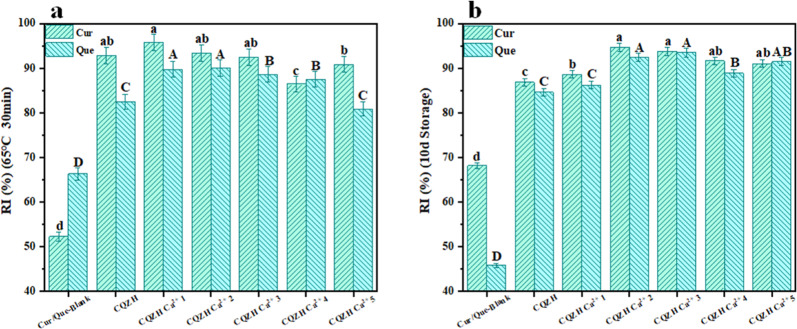
Stability analysis of samples after heat treatment and 10-day storage. Stability of Cur/Que before and after encapsulation of nanoparticles with pasteurization heat treatment (**a**) and 10-day storage (**b**), respectively. Values with different letters are statistically different (*p* < 0.05). Data were expressed as mean ± standard deviation.

Finally, the physicochemical stability of nutraceutical-loaded nanoparticles was measured because this is important for their practical application. For this reason, we monitored the resistance of curcumin and quercetin in the CQZH nanoparticles to chemical degradation after simulated pasteurization and long-term storage. The effect of pasteurization (65 °C, 30 min) on the thermal degradation of the two nutraceuticals is shown in Fig. 7a. After this heat treatment, the retention of quercetin and curcumin was only 52.3% and 66.3% in the control group and 92.9% and 82.6% in the composite nanoparticles, respectively. This effect may be attributed to the ability of encapsulation to separate quercetin and curcumin from the surrounding aqueous phase. Similar results have been reported in other studies^36^. As shown in Fig. 7b, the degradation of curcumin and quercetin during storage was significantly improved after they were encapsulated in the CQZH nanoparticles. Again, this effect may be because encapsulation partially isolates the nutraceutical molecules from the aqueous phase, where chemical degradation reactions typically occur more rapidly^31^.

**Fig. 8:**
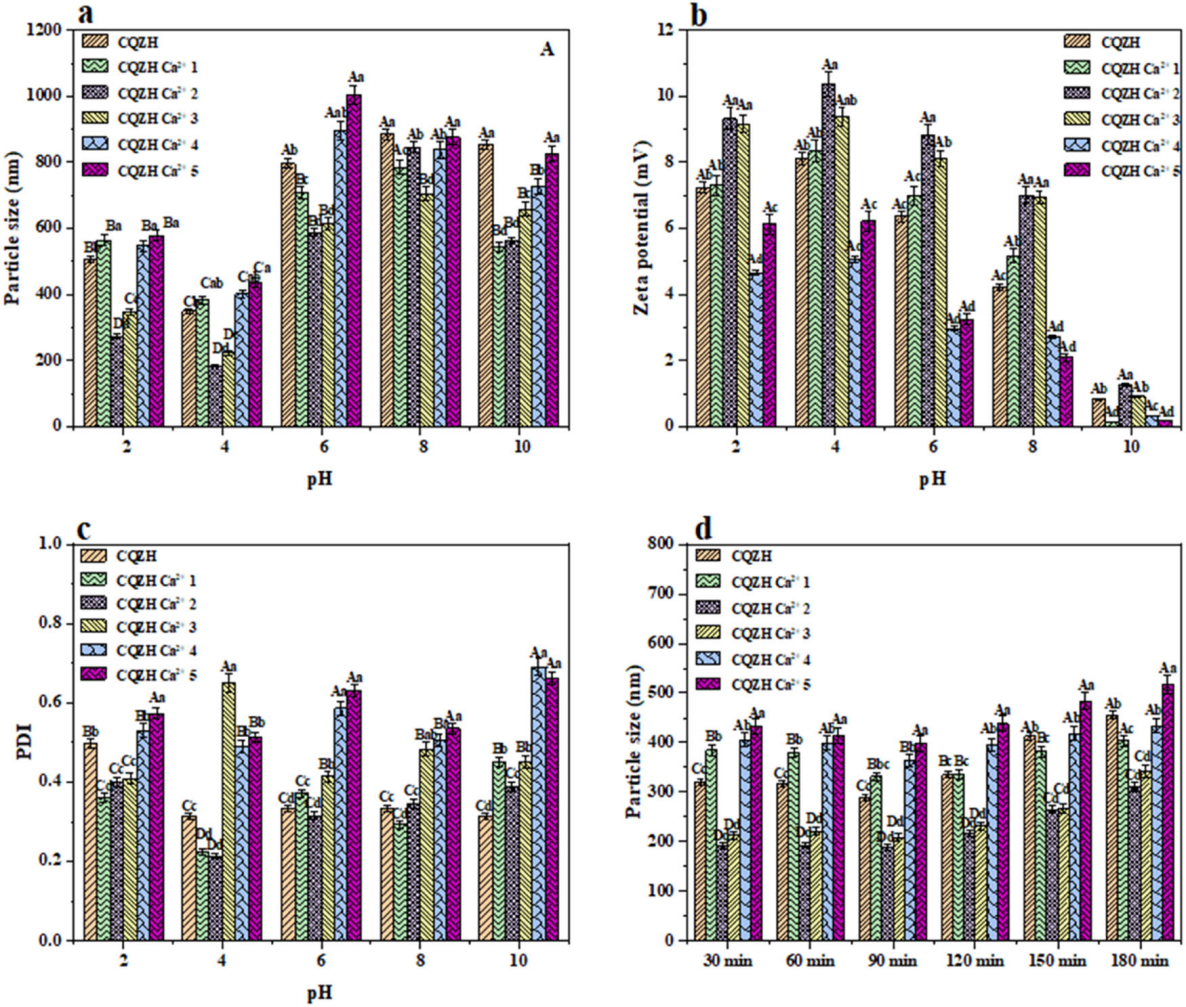
Effect of pH changes and in vitro simulated gastrointestinal digestion on sample stability. Effects of pH (**a**–**c**), and in vitro digestion (**d**) on the particle size of CQZH (different superscript letters (A (**a**), B (**b**), C (**c**)…) in the figure indicate significant differences (*p* < 0.05)). Data were expressed as mean ± standard deviation.

Nutraceutical-loaded nanoparticles must often remain stable when exposed to different pH conditions in food products and within the gastrointestinal tract. For this reason, we measured the impact of pH on the particle size, polydispersity, and charge of the CQZH nanoparticles (Fig. 8a–c). The magnitude of the positive charge on the nanoparticles was greatest at pH 4 but decreased when the pH was raised or lowered from this value, which would be expected to impact the electrostatic interactions in the system (Fig. 8b). Indeed, when the nanoparticle dispersions were adjusted from pH 4 to 2, the size of the particles increased, which was mainly attributed to aggregation caused by a reduction in electrostatic repulsion^20^. Similarly, when the nanoparticle dispersions were adjusted from pH 4 to 6 (and above), the size of the particles increased significantly, which was also attributed to aggregation caused by a reduction in electrostatic repulsion^37^. In addition, when the pH environment is near the isoelectric point, the net charge on the surface of the zein molecule is close to zero, and at this time, the protein molecular particles in the solution do not have the same charge to repel each other. The interaction between molecules is weakened, and the particles are easy to collide, condense, and precipitate. Therefore, the solubility of protein is at its minimum at the isoelectric point. Zein nanoparticles showed an obvious aggregation phenomenon, and the average particle size increased significantly^38^. In general, the CQZH nanoparticles containing calcium ions exhibited similar changes.

As shown in Fig. 8d, the particle size remained relatively stable after exposure to a simulated gastric fluid environment (30 and 60 min), which suggests that the CQZH nanoparticles are able to avoid pepsin degradation^20^. After entering the intestinal digestive environment (90, 120, 150, and 180 min), the size of the CQZH nanoparticles gradually increased, indicating that the structure of the CQZH nanoparticles was gradually altered under small intestine conditions. This is due to the fact that the pH environment and enzyme environment changed greatly when the nanoparticles entered the simulated intestinal fluid environment from the simulated gastric fluid environment. When the pH environment around the nanoparticles changes from 4.0 to 7.4, the size of the particles increases, which may be because the pH environment is closer to the isoelectric point of zein and the net charge on the surface of the particles decreases, resulting in the agglomeration of the particles^19^, which is consistent with the results shown in Fig. 8b. In addition, under the action of trypsin, zein hydrolyzed, the protein structure stretched, and the core-shell structure formed by the protein and cyclodextrin was damaged, which also affected the particle size change of the nanoparticles to a certain extent^20,39^. Similar results were reported by Wei et al.^20^ for the stability of curcumin-loaded zein-propylene glycol alginate composite nanoparticles during gastrointestinal digestion. The composite nanoparticles containing calcium exhibited a similar trend, but there were some differences depending on calcium concentration. For instance, the composite nanoparticles prepared with intermediate calcium concentrations (2–3 mM) had the smallest particle sizes throughout the simulated gastrointestinal tract.

## Discussion

In this study, we prepared nutraceutical-loaded composite nanoparticles with core–shell structures using a simple antisolvent co-precipitation method combined with an electrostatic deposition method. The effects of calcium ions on the properties of the nanoparticles and the stability and biological activities of encapsulated curcumin and quercetin were measured. Our results showed that the two nutraceuticals could be successfully encapsulated in the composite particles, which improved their resistance to pasteurization and extended their shelf life. The results of free radical scavenging experiments showed that the two hydrophobic polyphenols had synergistic antioxidant effects. Spectroscopic analysis showed that hydrogen bonding, hydrophobic, and electrostatic interactions played a key role in the assembly of the composite nanoparticles. X-ray diffraction analysis indicated that the interiors of the composite nanoparticles were amorphous rather than crystalline, which indicated that the hydrophobic polyphenol molecules were successfully incorporated into the protein matrix inside the nanoparticles. The composite nanoparticles were relatively resistant to pH changes and gastric conditions, but they were degraded under small intestine conditions, which would be important for the release of the nutraceuticals in the human gastrointestinal tract. Overall, the composite nanoparticles prepared in this study may be useful for the encapsulation, protection, and delivery of hydrophobic bio-active substances, thereby facilitating their efficacy in functional food and beverage products. However, their actual efficacy needs to be further investigated using in vivo animal and human studies, which will be the focus of our future research efforts.

## Methods

### Materials

Zein was obtained from Sigma-Aldrich (Shanghai, China). HP-β-CD was provided by Qianhui Biotechnology Co., Ltd. (Zibo, China). Curcumin and quercetin were purchased from Aladdin Biochemical Technology Co., Ltd. (Shanghai, China). All other reagents used were of analytical grade.

### Preparation of composite nanoparticles

The zein/HP-β-CD composite nanoparticles (CQZH) co-encapsulated with curcumin and quercetin were prepared using an antisolvent co-precipitation method combined with electrostatic interactions, as described previously^20^. Firstly, 2.0 g of zein, 100 mg of curcumin, and 100 mg of quercetin were dissolved in 200 mL of aqueous ethanol solution (75%, v/v) and stored overnight at 4 °C. Then, the mixed solution was slowly added dropwise to 3 times the volume of ultrapure water (pH = 4.0) with continuous stirring at 650 rpm to form a dispersion of Cur-Que-zein nanoparticles through antisolvent precipitation. The above dispersion was then added dropwise to 600 mL of HP-β-CD aqueous solution in the same manner with continuous stirring to obtain a CQZH composite particle dispersion. This dispersion was then incubated at 4 °C for 1 h, then 60 mL of calcium ion solution was dispersed into it. The ethanol was then removed from the resulting mixture using a rotary evaporator (35 °C, 0.1 MPa), and the sample was freeze-dried to obtain composite nanoparticles. Composite nanoparticles prepared using calcium ion concentrations of 1–5 mM are referred to as CQZH Ca^2+^1, CQZH Ca^2+^2, CQZH Ca^2+^3, CQZH Ca^2+^4, and CQZH Ca^2+^5, respectively.

### Characterization of nanoparticles

#### Particle size, ζ-potential, and morphology

The mean particle size, polydispersity index (PDI), and ζ-potential values of the samples were measured at 25 °C using an instrument that combines dynamic light scattering and particle electrophoresis (Nano-ZS Zetasizer, Malvern, UK), as described earlier. The morphology of the zein- HP-β-CD, CQZH, and CQZH Ca^2+^ nanoparticles were observed using field emission scanning electron microscopy (FE-SEM, SU8010, Hitachi)^40^.

#### Encapsulation properties

The encapsulation properties of the nanoparticle dispersions were determined by centrifuging them at 8000 rpm for 30 min to remove any insoluble material. The concentration of the two nutraceuticals was then determined by measuring the absorbance of diluted solutions using a UV spectrophotometer (Model UV-1800PC, Mapada, Shanghai, China). The encapsulation efficiency (EE) and loading capacity (LC) of the quercetin and curcumin in the nanoparticles was then determined using the method of Haider et al.^41^ The concentrations of quercetin (*C*_Que_) and curcumin (*C*_Cur_) were calculated from the absorbance (*A*) measurements using suitable calibration curves: *A*_Que_ = 72.564C_Que_ (*R*^2^ = 0.9998); *A*_Cur_ = 0.2478C_Cur_ + 0.0012 (*R*^2^ = 0.9999). The EE and LC values of the two nutraceuticals were then calculated using the following equations:

1 $${{{\mathrm{EE}}}}\left( \% \right) = \frac{{{{{C}}}_{\rm{Total}} - C_{\rm{Free}}}}{{{{{C}}}_{\rm{Total}}}} \times 100$$

2 $${{{\mathrm{LC}}}}\left( \% \right) = \frac{{{{{C}}}_{\rm{Total}} - {{{C}}}_{\mathrm{Free}}}}{{{{{C}}}}_{\mathrm{Complex}}} \times 100$$

Here, *C*_Total_ and *C*_Free_ are the total and free concentrations of the nutraceuticals in the systems, and *C*_Complex_ is the concentration of the complexes in the system (nanoparticles + nutraceuticals).

#### FT-IR, XRD, and thermogravimetric (TG) analysis

The freeze-dried samples were analyzed using an FT-IR spectrometer (Nicolet Nexus 470, United States). Samples were prepared using the potassium bromide tablet method and then scanned from 4000 to 400 cm^−1^, as described previously^15^.

The crystal structure of the lyophilized samples was characterized using an X-ray diffraction instrument (D2 PHASER, Bruker, Germany), as described previously^42,43^. The test conditions used were accelerating voltage = 40 kV; current = 40 mA; step size = 0.05; test speed = 0.05 s/step; and scanning range (2*θ*) = 4–40°.

The thermal stability of the lyophilized samples was tested using a thermogravimetric analyzer (TGA2, Mettler–Toledo, Schwerzenbach, Switzerland), as described previously^41^. The test conditions used were sample size = 3–4 mg; temperature range = 30–600 °C; heating rate = 10 °C/min; and nitrogen flow rate = 20 mL/min.

#### Fluorescence, ultraviolet, and circular dichroism spectroscopy

The fluorescence spectra of the samples were acquired using a fluorescence spectrophotometer (F-7000, Hitachi, Tokyo, Japan), as described previously^44^. Prior to analysis, the composite nanoparticles were diluted to 0.1 mg/ml with ultrapure water. The excitation wavelength used was 280 nm, and the emission wavelength range was 290–50 nm. Simultaneous fluorescence spectra were recorded at constant wavelength intervals (Δλ = 15 nm and Δλ = 60 nm) by simultaneous scanning of the excitation and emission monochromators.

The ultraviolet–visible spectra of the samples were acquired using a UV spectrophotometer (UV-2600, Shimadzu Scientific Instruments, Kyoto, Japan), as described previously^45^. Samples were placed in cuvettes and then scanned from 200 to 700 nm.

Circular dichroism analysis was carried out using a CD spectrophotometer (Applied Photophysics Ltd., Surrey, UK), as described previously^20^. The far-UV wavelength range for CD measurements was set to 190–260 nm. The measurement parameters used were bandwidth = 1.0 nm, step = 1.0 nm, time-per-point = 0.5 s, resolution = 0.1 cm, and the number of points accumulated = 20.

### DPPH radical scavenging activity

The sample dispersion was mixed with DPPH-ethanol solution (0.1 mM) in equal volume and placed in a dark place for 30 min. After the reaction was completed, the absorbance (*A*_t_) of the mixed solutions was measured at 517 nm, as described elsewhere^19^. In addition, the absorbance of the ethanol-substituted DPPH-ethanol solution (*A*_b_), and deionized water-substituted sample (*A*_c_) were also recorded. The free radical scavenging rate of the tested samples was then derived from the following expression:

3 $${{{\mathrm{DPPH}}}}\,{{{\mathrm{scavenging}}}}\,{{{\mathrm{ability}}}}\left( \% \right) = \left( {1 - \frac{{{{{A}}}_{{{\mathrm{t}}}} - {{{A}}}_{{{\mathrm{b}}}}}}{{{{{A}}}_{{{\mathrm{c}}}}}}} \right) \times 100$$

### Storage and heat stability of composite NPs

The impact of calcium ion concentration on the resistance of the nutraceutical-loaded nanoparticles to changes in environmental conditions was measured, as described previously^44^. The thermal stability of the nanoparticles was determined by exposing the samples to low-temperature pasteurization conditions (65 °C, 30 min). The storage stability of the nanoparticles was determined by storing them under ambient conditions for 10 days. The residual amounts of curcumin and quercetin in the formulations were obtained by measuring the absorbance at 428 and 374 nm, respectively. The retention index (RI) was then calculated as follows:

4 $${{{\mathrm{RI}}}}\left( \% \right) = \frac{{{{{C}}}_{\rm{R}}}}{{{{{C}}}_{\rm{I}}}} \times 100$$

Here *C*_R_ and *C*_I_ are the remaining and initial concentrations of the nutraceuticals in the composite nanoparticles, respectively (The sample concentration was calculated using the calibration curve described in the Section of “Encapsulation properties”).

### Impact of pH and simulated gastrointestinal conditions on nanoparticles

The nutraceutical-loaded nanoparticles were adjusted to a range of pH values (pH 2–10) using NaOH or HCl solutions, and then the particle size and zeta potential values were measured after they were stored for 18 h at 25 °C^46^. The influence of simulated gastrointestinal conditions on the stability of the nutraceutical-loaded nanoparticles was assessed using an in vitro digestion model described earlier^47^. The nanoparticle dispersion was first diluted in simulated gastric juice (1 mg/mL pepsin, pH 4.0), followed by digestion for 1 h (37 °C). The resulting system was then diluted in a simulated small intestine solution (4 mg/ml trypsin, pH 7.4) and digested for 2 h (37 °C). Samples were taken every 30 min to determine the change in particle size and zeta potential.

### Statistical analysis

Average values were calculated from three parallel experiments, and statistics were carried out using SPSS software for variance analysis, and *p* < 0.05 is considered statistically significant.

### Reporting summary

Further information on research design is available in the Nature Research Reporting Summary linked to this article.

## Supplementary information

Supplementary data

Reporting Summary

## Data Availability

The authors declare that all data supporting the findings of this study are available in the paper.
