# Supplementary material for: Co-encapsulation of curcumin and quercetin with zein/HP-β-CD conjugates to enhance environmental resistance and antioxidant activity
Source: NPJ Sci Food. 2023 Jun 14;7:29. doi: 10.1038/s41538-023-00186-2 (PMC10267186; doi:10.1038/s41538-023-00186-2)
Supplement: Supplementary file 1 — Supplementary data [file 41538_2023_186_MOESM1_ESM.pdf]

Supplementary Data

Co-encapsulation of curcumin and quercetin with zein/HP-β-CD conjugates to enhance environmental resistance and antioxidant activity

Chao Qiu[1,*], Zhiheng Zhang[1,*], Xiaojing Li[2], Shangyuan Sang[3], David Julian McClements[4], Long Chen[1], Jie Long[1], Aiquan Jiao[1], Xueming Xu[1], Zhengyu Jin[1,**]

[1] State Key Laboratory of Food Science and Technology, School of Food Science and Technology, Jiangnan University, Collaborative innovation center of food safety and quality control in Jiangsu Province, Wuxi, Jiangsu 214122, China

[2] College of Light Industry and Food Engineering, Nanjing Forestry University, Jiangsu 210037, China

[3] Zhejiang-Malaysia Joint Research Laboratory for Agricultural Product Processing and Nutrition, Key Laboratory of Animal Protein Food Deep Processing Technology of Zhejiang Province, College of Food and Pharmaceutical Sciences, Ningbo University, Ningbo 315832, China

[4] Department of Food Science, University of Massachusetts (Amherst, Massachusetts 01060, United States)

*These authors contributed equally to this work and should be considered co-first authors.

***Corresponding author*: Prof. Zhengyu Jin (Tel:/Fax: 86-51085913299; Email: fpcenter@jiangnan.edu.cn).

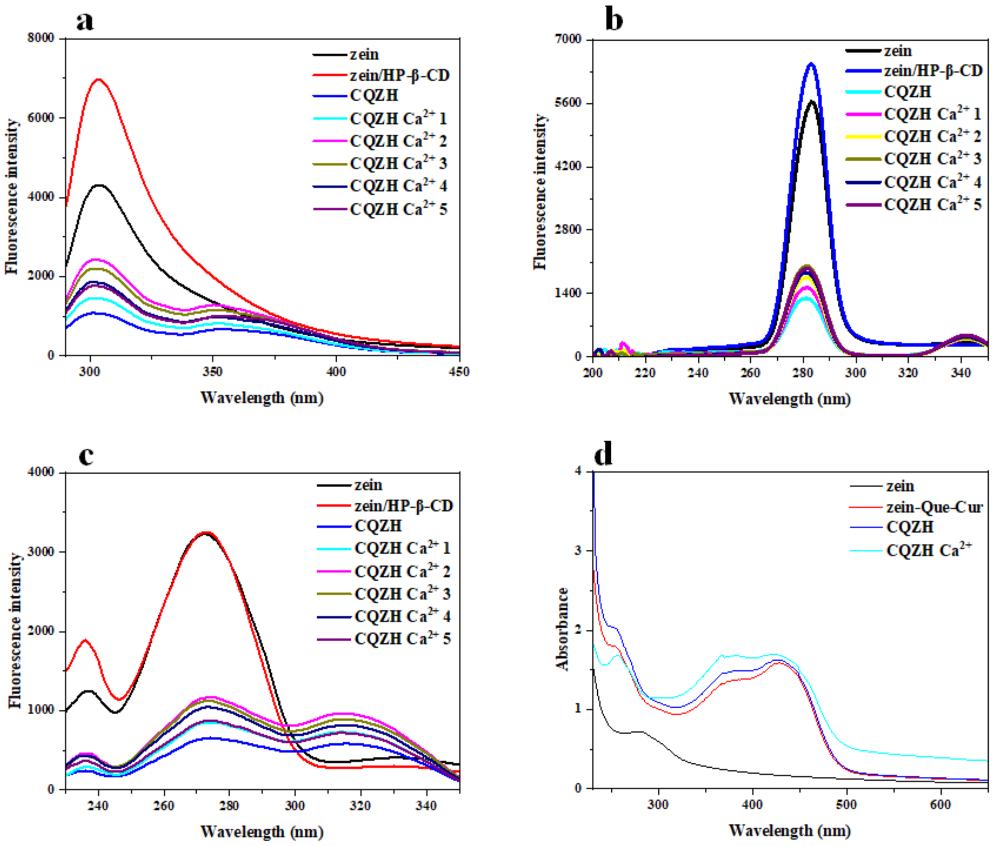

Supplementary Figure. 1. Fluorescence absorption spectra (a), synchronous fluorescence spectra (b: Δλ= 15 nm, c: Δλ= 60 nm)), and UV-vis absorption spectra (d) of zein, zein/HP-β-CD, CQZH, and CQZH Ca^{2+} (1–5 mM Ca^{2+}).

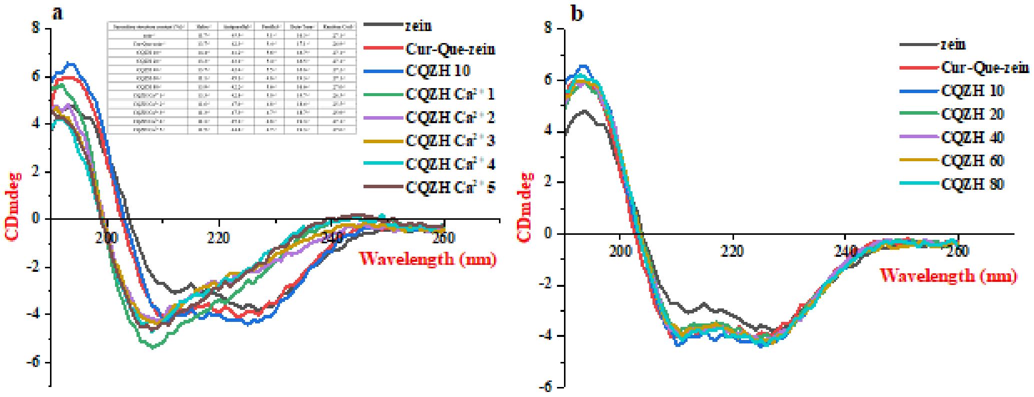

Supplementary Figure. 2. Effect of calcium addition on secondary structure of zein (a); Effect of cyclodextrin addition on secondary structure of zein in CQZH (b) (CQZH 10, 20, 40, 60, and 80 means that the sample was supplemented with curcumin and quercetin at a concentration of 10 μg/mL, 20 μg/mL, 40 μg/mL, 60 μg/mL, 80 μg/mL, respectively).
